# Supplementary material for: Prevalence of elevated liver transaminases and their relationship with alcohol use in people living with HIV on anti-retroviral therapy in Uganda
Source: PLoS One. 2021 Jun 1;16(6):e0250368. doi: 10.1371/journal.pone.0250368 (PMC8168875; doi:10.1371/journal.pone.0250368)
Supplement: S1 File — (PDF) [file pone.0250368.s001.pdf]

ADEPTT Study Screening Step 1

Initial Eligibility Screening – self-report

DATE: __ __ / __ __ / __ __ __ __ (DAY/MONTH/YEAR)

ADEPTT SCREENING ID: SCT __ __ __ __

Completed by phone or in-person?	☐ **Phone**	☐ **In-person**
Is this a Uganda ARCH prior participant?	☐ **Yes**	☐ **No**
If prior participant, enter study ID:<br>ADEPT Study ID: MBD __ __ __ __	BREATH Study ID: MBB __ __ __ __	
What is the patient's sex?	☐ **Male**	☐ **Female**

Initial eligibility assessment.

1. How old are you? __ __ **years** → is patient 18 years or older?	☐ **Yes**	☐ No
2. Are you fluent in either Runyakole or English?	☐ **Yes**	☐ No
3. Have you been on ART for 6 or more months?	☐ **Yes**	☐ No/Don't know
3a. If yes: Are you currently on or prescribed Nevirapine (NVP)?	☐ Yes	☐ **No**
3b. If not currently on NVP: Have you taken NVP in the past 2 weeks?	☐ Yes	☐ **No**
4. Do you live within 2 hours travel time of the ISS Clinic?	☐ **Yes**	☐ No
5. Do you have plans to move more than 2 hours from the ISS Clinic, within the next 6 months?	☐ Yes	☐ **No**
6. Have you ever had active TB before?	☐ Yes	☐ **No/Don't know**
7. Have you ever taken TB medications before, to treat or prevent TB?	☐ Yes	☐ **No/Don't know**
8. Are you currently taking anti-convulsion medications, or do you have any plans to take them in the future?	☐ Yes	☐ **No**
9. Have you had any alcohol to drink in the past year?	☐ **Yes**	☐ **No**
9a. If yes: Have you had any alcohol to drink in the past 3 months?	☐ **Yes**	☐ No

Eligible: **YES** to questions 1, 2, 3 & 4; **NO/DON'T KNOW** to questions 5, 6, 7 & 8. (ticks in the unshaded boxes)
Ineligible: Currently on NVP (**YES** to 3a) or taken NVP in the past 2 weeks (**YES** to question 3b), or Prior year drinker who is not a prior 3 month drinker, but who drank 4-12 months ago (ie. **YES** to question 9 *plus* **NO** to question 9a) (ticks in a shaded box)

If eligible: ask participant if they are interested in continuing with further screening.

Eligibility status:

☐ Ineligible.

☐ Eligible, declines further screening. **(specify reason to the right)**

☐ Eligible. **(Refer to RA for consent for further screening)**

Reason for declining further screening:

☐ 1 = Time barred

☐ 2 = Stigma/disclosure issues

☐ 3 = Needs additional approval from family member

☐ 4 = Too weak

☐ 5 = Not interested

☐ 6 = Declines blood draw

☐ 7 = Declines to answer

☐ 8 = Other (specify) ________________________

Notes:

Screener initials: ____ ____

	Initials	Date
QC check		
Entry 1		
Entry 2		
