# Supplementary material for: Prevalence of elevated liver transaminases and their relationship with alcohol use in people living with HIV on anti-retroviral therapy in Uganda
Source: PLoS One. 2021 Jun 1;16(6):e0250368. doi: 10.1371/journal.pone.0250368 (PMC8168875; doi:10.1371/journal.pone.0250368)
Supplement: Attachment — Submitted filename: Response to Reviewers.docx [file pone.0250368.s002.docx]

PONE-D-20-39571
Prevalence of Elevated Liver Transaminases and Their Relationship with Alcohol Use in People Living with HIV on Anti-Retroviral Therapy in Uganda
PLOS ONE

Dear Ms. Perez and Dr. Chemin,

Thank you for your review of out manuscript. Rebuttals to your questions are provided below.

1. Please ensure that your manuscript meets PLOS ONE's style requirements, including those for file naming.

*We have done so.*

2. We note that you have stated that you will provide repository information for your data at acceptance. Should your manuscript be accepted for publication, we will hold it until you provide the relevant accession numbers or DOIs necessary to access your data. If you wish to make changes to your Data Availability statement, please describe these changes in your cover letter and we will update your Data Availability statement to reflect the information you provide.

*The data are available at* [https://datadryad.org/stash/share/Tp491NLcUe8qTcDADYiI8RtiuIFiq9lm6RAOm9zbvBI](https://urldefense.proofpoint.com/v2/url?u=https-3A__datadryad.org_stash_share_Tp491NLcUe8qTcDADYiI8RtiuIFiq9lm6RAOm9zbvBI&d=DwMFaQ&c=iORugZls2LlYyCAZRB3XLg&r=CM5-M9qwH6M7BJpCz1wxz3ziCdVoQEIFczws1Bsi08U&m=ynMR5lMh0T_FuYzEeyIfXNziopRV6qAvC2X2L_jKxfI&s=zN2h6BLaCreyEtlyrVonjf-xPrxgl15WSpZF0SAPtQU&e=)*DOI#* 10.7272/Q66H4FPZ

3.Thank you for stating the following in the Competing Interests section:

"Potential conflict of interest:
Dr. Debbie Cheng serves on Data Safety and Monitoring Boards for Janssen. Her work for Janssen is unrelated to the submitted work and Janssen had no role in any aspect of this work."

*This does not alter our adherence to PLOS ONE policies on sharing data and materials.*

4. We note that you have included the phrase “data not shown” in your manuscript. Unfortunately, this does not meet our data sharing requirements.

*This was in error. These data are included in the above-mentioned file. We meant to note that we were not including a separate table for the analyses, but the results were presented iwhtin the paragraph. We have removed the phrase from the manuscript.*

5. Please include additional information regarding the survey or questionnaire used in the study and ensure that you have provided sufficient details that others could replicate the analyses. For instance, if you developed a questionnaire as part of this study and it is not under a copyright more restrictive than CC-BY, please include a copy, in both the original language and English, as Supporting Information, or include a citation if it has been published previously.

*We have included the screening form, that collected participant age, sex, and current alcohol use as file S1.*

6. In the Methods, please discuss whether and how the questionnaire was validated and/or pre-tested. If these did not occur, please provide the rationale for not doing so.

*The only data from a questionnaire were from the standardized AUDIT-C scale, which is referenced in the text.*

As requested on March 1:

*All participants provided written informed consent.*

Reviewers' comments:

Reviewer #1: Assuming the sample is appropriate, this is a straight forward descriptive statistical application of the use of multiple logistic regression models and calculated odds ratios (OR) and 95% confidence intervals (CI), on an imputed sample. It appears that only about 7 percent of the sample had to be imputed. The results appear to follow from the analysis presentation of Table 2, in that current drinking and male sex were independently associated with elevated transaminases.

*No edits are needed in response to this comment.*
